# Changes in Plasma von Willebrand Factor and Cellular Fibronectin in MRI-Defined Traumatic Microvascular Injury

**DOI:** 10.3389/fneur.2019.00246

**Published:** 2019-03-26

**Authors:** Danielle K. Sandsmark, Tanya Bogoslovsky, Bao-Xi Qu, Margalit Haber, Martin R. Cota, Cora Davis, John A. Butman, Lawrence L. Latour, Ramon Diaz-Arrastia

**Affiliations:** ^1^Department of Neurology, University of Pennsylvania, Philadelphia, PA, United States; ^2^Division of Clinical Neurosciences, Turku University Hospital, University of Turku, Turku, Finland; ^3^Center for Neuroscience and Regenerative Medicine, Bethesda, MD, United States; ^4^Acute Cerebrovascular Diagnostics Unit, National Institute of Neurological Disorders and Stroke, Bethesda, MD, United States; ^5^Department of Neurology, Uniformed Services University of the Health Sciences, Bethesda, MD, United States; ^6^National Institutes of Health, Radiology and Imaging Sciences, Bethesda, MD, United States

**Keywords:** TBI, vascular, MRI, biomarkers, trauma

## Abstract

The neuropathology of traumatic brain injury (TB) is diverse, including primary injury to neurons, axons, glial cells, vascular structures, and secondary processes, such as edema and inflammation that vary between individual patients. Traumatic microvascular injury is an important endophenotype of TBI-related injury. We studied patients who sustained a TBI requiring ER evaluation and had an MRI performed within 48 h of injury. We classified patients into 3 groups based on their MRI findings: (1) those that had evidence of traumatic microvascular injury on susceptibility or diffusion weighted MRI sequences without frank hemorrhage [Traumatic Vascular Injury (TVI) group; 20 subjects]. (2) those who had evidence of intraparenchymal, subdural, epidural, or subarachnoid hemorrhage [Traumatic Hemorrhage (TH) group; 26 subjects], and (3) those who had no traumatic injuries detected by MRI [MRI-negative group; 30 subjects]. We then measured plasma protein biomarkers of vascular injury [von Willebrand Factor (vWF) or cellular fibronectin (cFn)] and axonal injury (phosphorylated neurofilament heavy chain; pNF-H). We found that the TVI group was characterized by decreased expression of plasma vWF (*p* < 0.05 compared to MRI-negative group; *p* < 0.00001 compared to TH group) ≤48 h after injury. cFN was no different between groups ≤48 h after injury, but was increased in the TVI group compared to the MRI-negative (*p* < 0.00001) and TH (*p* < 0.00001) groups when measured >48 h from injury. pNF-H was increased in both the TH and TVI groups compared to the MRI-negative group ≤48 h from injury. When we used the MRI grouping and molecular biomarkers in a model to predict Glasgow Outcome Scale-Extended (GOS-E) score at 30–90 days, we found that inclusion of the imaging data and biomarkers substantially improved the ability to predict a good outcome over clinical information alone. These data indicate that there is a distinct, vascular-predominant endophenotype in a subset of patients who sustain a TBI and that these injuries are characterized by a specific biomarker profile. Further work to will be needed to determine whether these biomarkers can be useful as predictive and pharmacodynamic biomarkers for vascular-directed therapies after TBI.

## Introduction

TBI is one of the leading causes of death and disability around the world (1). Recent epidemiological studies indicate that around 5.3 million people in the United States live with a TBI-related disability (2). One of the primary challenges in TBI care is the inability to accurately identify those at risk for chronic functional impairments in the early period after their injury (3). Current classification schemes label injuries as mild, moderate, or severe based on clinical or demographic features, including age, duration of loss of consciousness, and presence of hemorrhage on head CT, but do not consider the underlying pathology associated with the injury. Beyond clinical neurological assessments and head CT imaging, there are no imaging or molecular biomarkers that reliably reflect the underlying neuropathology or prospectively identify patients who may benefit from biologically-relevant therapies (4, 5). The neuropathology of TBI is complex, including axonal shearing, inflammation, brain edema, and vascular injury, but the extent of these findings varies significantly between individual patients (6–10). Conventional imaging studies, including computed tomography (CT) and magnetic resonance imaging (MRI) do not reliably capture the full extent of the injury, particularly in those patients with mild injuries (11). As a result, current classification tools are most helpful for predicting survival in those patients with the most severe injuries but are generally not useful for predicting functional outcomes in longer term survivors or those with milder injuries (4, 5). The lack of available tools to assist in diagnosis and prognosis after TBI has led to a call for biomarkers that correlate with these varied pathologies, track with disease recovery, and predict long-term outcomes (12). The availability of such biomarkers would facilitate accurate TBI phenotyping to allow more appropriate selection of targeted therapies and facilitate monitoring of therapeutic response.

Of the various pathologies characteristic of TBI, cerebrovascular injury is an important but relatively understudied endophenotype of TBI-related brain injury (13). Traumatic microvascular injury (TVI) is a common finding both in human TBI and in preclinical animal models, including mild and repetitive TBI (6, 9, 13). In humans, examination of pathological specimens demonstrates that TVI is a prominent finding in patients who die in the first few days following TBI, even after relatively mild brain injuries (14, 15). Neuroimaging biomarkers of TVI include both head CT and MRI. In the most severe injuries, there is obvious traumatic hemorrhage or contusion that is visible on head CT. More subtle microvascular injury, due to disruption of precapillary arterioles and capillaries, can be identified using diffusion weighted imaging (DWI), gradient echo (GRE), susceptibility weighted (SWI) MRI sequences. DWI is very sensitive to tissue ischemia (16). The burden of DWI+ lesions has been associated with neurological outcomes in adult and pediatric TBI (16, 17). GRE and SWI sequences rely on the magnetic properties of iron in blood hemosiderin to identify regions of acute or chronic bleeding (18–20). These MRI-identified traumatic hemorrhages are most frequently observed in cortical gray matter, subcortical white matter, or within major white matter tracts (corpus callosum, brainstem, or internal capsule). Small venous hemorrhages that take on a flame or linearly shaped appearance can be seen even in the mildest forms of TB (13, 20, 21), but are not present in non-traumatic acute brain injury (21), indicating that these lesions reflect local strain that disrupts the vascular elements. These small venous injuries are often in close proximity to areas of axonal injury identified by diffusion tensor imaging (20). This has led to confusing nomenclature, as MRI-identified microhemorrhages are often referred to as “diffuse axonal injury” although they do not measure changes in axonal function or connectivity (20, 22). The presence of SWI lesions has been associated with poorer neurological and functional outcomes after TBI (23, 24).

In this study, we reviewed radiological data of MRIs performed within 48 h following TBI. From this group of acutely injured TBI patients, we identified groups of TBI subjects who had structurally normal MRIs, those who had evidence of traumatic intracranial hemorrhages/contusions in the brain parenchyma, subdural, or subarachnoid spaces (“traumatic hemorrhage (TH) group”), and those who had MRI evidence of microvascular injury without frank hemorrhage (“TVI group”). We compared these imaging findings with circulating protein biomarkers of vascular injury, von Willebrand factor (vWF) and cellular fibronectin (cFN). vWF is a large, multimeric protein important for thrombus formation at the site of vascular injury and is a known biomarker of systemic endothelial injury. Elevated levels of circulating vWF following head injury have been associated with poorer neurological outcomes following severe head trauma (25, 26). cFN is released by pericytes and serves as a marker of blood-brain barrier integrity. Elevated plasma levels of cFN have been associated with poorer outcomes after severe TBI (27) We measured vWF and cFN along with phosphorylated neurofilament heavy chain (pNF-H), a biomarker of axonal injury (28, 29). We show that TBI patients with different MRI characteristics also exhibit different biomarker profiles. vWF is elevated in the TH group compared to the MRI-negative group, while the TVI group had lower levels of vWF. There was no difference between the groups in cFN within the first day of injury, but cFN was elevated in the TVI group at later time points. pNF-H was elevated in both TH and TVI groups in the first 24 h after injury, but remained elevated only in the TVI group. Thus, these 3 MRI-defined groups exhibit distinct biomarker profiles. When incorporated into predictive models, biomarker expression aided in predicting good outcome beyond what MRI findings and clinical/demographic information could provide. While these results require confirmation in a larger clinical cohort, they suggest that plasma vWF and cFN may be useful predictive biomarkers in TBI.

## Methods

### Subject Recruitment and Enrollment

This study was reviewed and approved by the appropriate human patient protection authorities at the National Institutes of Health, Uniform Services University of the Health Sciences, Johns Hopkins Suburban Hospital, and MedStar Washington Hospital Center. All patients or surrogates provided informed consent before any study procedure. Patients were enrolled in the Center for Neuroscience and Regenerative Medicine (CNRM) Traumatic Head Injury Neuroimaging Classification protocol (NCT01132937) if they sustained a head injury and presented within 48 h of the event to the emergency department of MedStar Washington Hospital Center (Washington, DC) or Johns Hopkins Suburban Hospital (Bethesda, MD), level 1 and level 2 trauma centers, respectively. Study participants were enrolled to have a research MRI performed within 48 h of injury (21) using the following criteria: (1) high clinical suspicion of non-penetrating acute TBI, determined by the ED physician, for which a head CT scan was performed, (2) age ≥18 years, (3) interval between time of injury and enrollment <48 h, (4) and ability to obtain informed consent from the subject or a legally authorized representative. Potential subjects were excluded if they were (1) considered to be psychiatrically unstable by the patient's attending physician, (2) unable to have an MRI scan due to implanted, MRI-incompatible devices, (3) unable to enter the MRI scanner due to morbid obesity or claustrophobia, or (3) pregnant. All patients (or their legally authorized representatives) gave written informed consent for the use of participant's clinical data and blood samples in TBI research in accordance as approved by the Institutional Review Board. The Glasgow Coma Scale (GCS), post-traumatic amnesia, relevant injury and patient history, and clinical diagnoses were recorded and captured on standardized case report forms by the research team. Both blood sample and de-identified demographic, clinical, and radiological data were provided to the Center for Neuroscience and Regenerative Medicine (CNRM) Biorepository and approval was obtained for use for this project.

### Neuroimaging

All subjects obtained non-contrast head computed tomography scans as part of their clinical care. The clinical CT scans were interpreted by staff radiologists at the participating hospitals on admission and the clinical reports were reviewed by study personnel and findings related to trauma were recorded. MRI scans were performed on a 1.5 T MR scanner (Sigma, General Electric, Milwaukee, WI) or a 3T (Philips Medical Systems, Amsterdam, The Netherlands) scanner. A subset of the sequences performed were analyzed in this study including: pre- and post-contrast fluid inversion recovery (FLAIR), T2^*^-weighted imaging using both conventional GRE and susceptibility weighted imaging (SWI), isotropic diffusion weighted imaging (DWI), and apparent diffusion coefficient (ADC) maps derived from the 15 direction *b* = 1,000 diffusion tensor imaging. All MRI scans were rated by a clinical radiologist and two scientists experienced in neuroradiology who evaluated the images for the presence of radiological findings associated with acute brain injury. Scans were scored for GRE, FLAIR, DWI, and SWI-positive findings based both on the number, size, and location of lesions using a standard scoring sheet used to grade all scans (21). Image characteristics were reported in a standardized reporting form. On DWI, images were read for: (1) punctate lesion defined as a hyperintense lesion <1 cm (2) multifocal lesions described as areas of hyperintense lesions distributed throughout the brain and (3) any hyperintense lesion >1 cm. On GRE or SWI sequences, images were read for: (1) microbleeds defined as punctate areas of hypointensity contiguous in no more than two slices and distributed throughout the brain; (2) linear hypointensities defined as areas of hypointensity contiguous in more than two adjacent slices; (3) intraparenchymal hematoma (> 1 cm); (4) extra-axial blood such as epidural hematoma (EDH) and subdural hematoma (SDH); (5) subarachnoid hemorrhage (SAH); and (6) intraventricular hemorrhage. On FLAIR, images were read for: (1) dural enhancement of meninges determined by comparison of pre- and post-contrast FLAIR, and defined as a focal meningeal hyperintensity after injection of contrast on post-contrast sequences; (2) SAH defined as a hyperintense signal in the subarachnoid space on both pre- and post-contrast FLAIR sequences; and (3) presence of edema. Imaging features that had been recorded in the database were extracted for this analysis.

A group of 20 subjects had predominantly MRI lesions consistent with TVI (TVI group) defined as: (1) presence of single or multiple (>5) microbleeds on GRE or SWI (<1 cm), (2) presence of hypointense branching or linear structures on SWI, (3) presence of single or multiple punctate hyperintensities (<1 cm) on DWI, (4) absence of major epidural hematoma (EDH), subdural hematoma (SDH), subarachnoid hemorrhage (SAH), or intraparenchymal hematoma (IPH) >1 cm on MRI. Twenty-six TBI subjects [Traumatic Hemorrhage (TH) group] had predominantly TBI-related hemorrhages (intraparenchymal hematomas >1 cm, SDH, SAH, and other imaging findings on DWI, SWI, or FLAIR attributable to TBI. Thirty TBI patients did not have imaging findings on TBI on MRI (MRI-negative group).

### Blood Sampling

**Figure 1:**
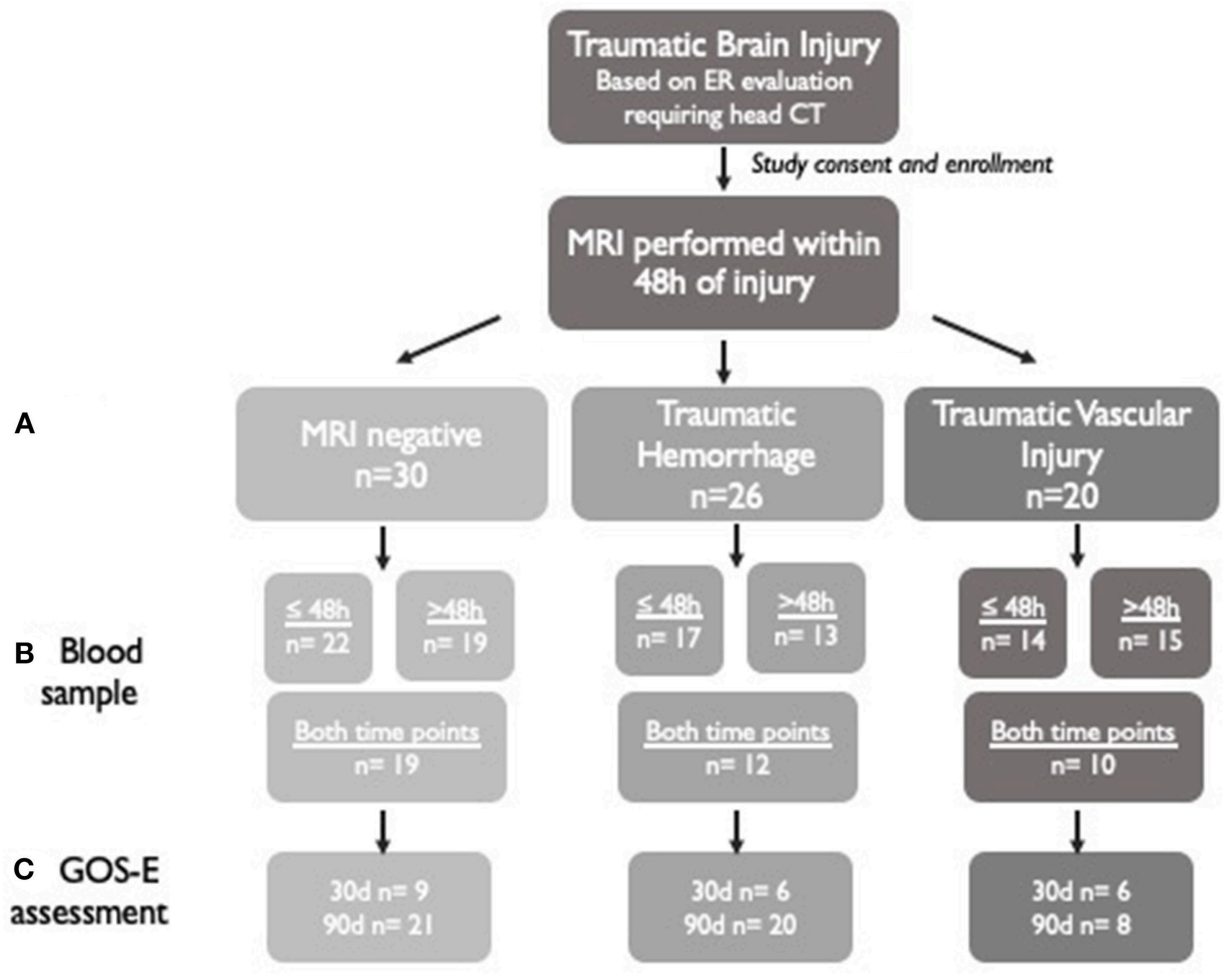
Schematic of study enrollment and available data. A total of 76 subjects who underwent brain MRI within 48 h of TBI were included in this study. **(A)** The subjects were divided into three groups based on MRI characteristics: MRI-negative, traumatic hemorrhage, and traumatic vascular injury. **(B)** Biosamples were collected at ≤48 h after injury and >48 h after injury if possible. Biosamples were collected at both time points on some patients. **(C)** Outcomes were assessed using the GOS-E administered at 30 or 90 days in subjects who were available for a study visit or telephone questionnaire. The highest GOS-E score attained by the participant at either time point was used in the analysis.

Non-fasting blood samples were collected <48 h from injury (median 20.7 h; IQR 10–29.4 h). Follow up blood samples were also collected from 48 h to 7 days after injury (median 136.7 h; IQR 63.3–182.1 h). When possible, blood was collected from the same subjects at both timepoints (Figure 1). Blood was collected into plastic dipotassium EDTA tubes, immediately placed on ice, centrifuged (15 min × 2,000 g) and frozen in aliquots within 3 h of collection. Samples were stored at −80°C in the CNRM Biorepository until time of analysis.

### Immunoassays

The plasma levels of biomarkers were measured using the enzyme-linked immunosorbent assay (ELISA) according to the manufacturer's instructions: pNF-H (BioVendor, Brno, Czech Republic) with limit of detection (LD) 23.5 pg/ml, intra- assay variability (CV) 4.5%), vWF (Life Technologies, Carlsbad, CA), with LD 0.4 ng/mL and interassay CV <10% and fibronectin (ThermoFisher, Waltham, MA) with LD 0.06 ng/mL and interassay CV <10%. The analyses were performed in a blinded fashion to MRI results and biosamples from the 3 groups were distributed between plates to minimize batch effects.

### Clinical Outcomes

The primary outcome measure used for this observational study was Glasgow Outcome Scale Extended score (GOS-E) (30). The GOS-E was collected in person or over telephone interview by study personnel. Collection was focused to include outcomes related to head injury, excluding any disability related to other injury in subjects, for the purposes of this study. GOS-E was determined between 30 and 90 days after injury and was available for 30 subjects in the MRI-negative group, 26 patients in the TH group, and 14 patients in the TVI group (Figure 1). If GOS-E was available from multiple timepoints after injury, the best score (highest functional outcome) was used in the analysis.

### Statistics

Descriptive statistics with frequencies and proportions were used to describe categorical variables. Biomarker levels were treated as continuous data. The Kruskal-Wallis test was used for continuous variables. Intracranial lesions shown on initial MRI were scored and analyzed as categorical variables. We assessed the ability of each biomarker level to discriminate patients in the MRI-negative, TH and TVI groups. Spearman correlation and linear regression were used to test the relationship between variables. Logistic regression was used to test the association of biomarkers with clinical outcomes, which were dichotomized as either good recovery (GOSE 7-8) or moderate-severe disability (GOSE ≤6). Nagelkerke and Hosmer-Lemeshow tests were used to describe the goodness of fit in the linear regression models. Statistical analysis was done using GraphPad Prism (v. 6.02; Graph Pad Software, San Diego, CA) or with Statistical Package for the Social Sciences SPSS (version 24, IBM Corporation, Armonk, NY).

## Results

### Demographics and Clinical Characteristics

**Figure 2:**
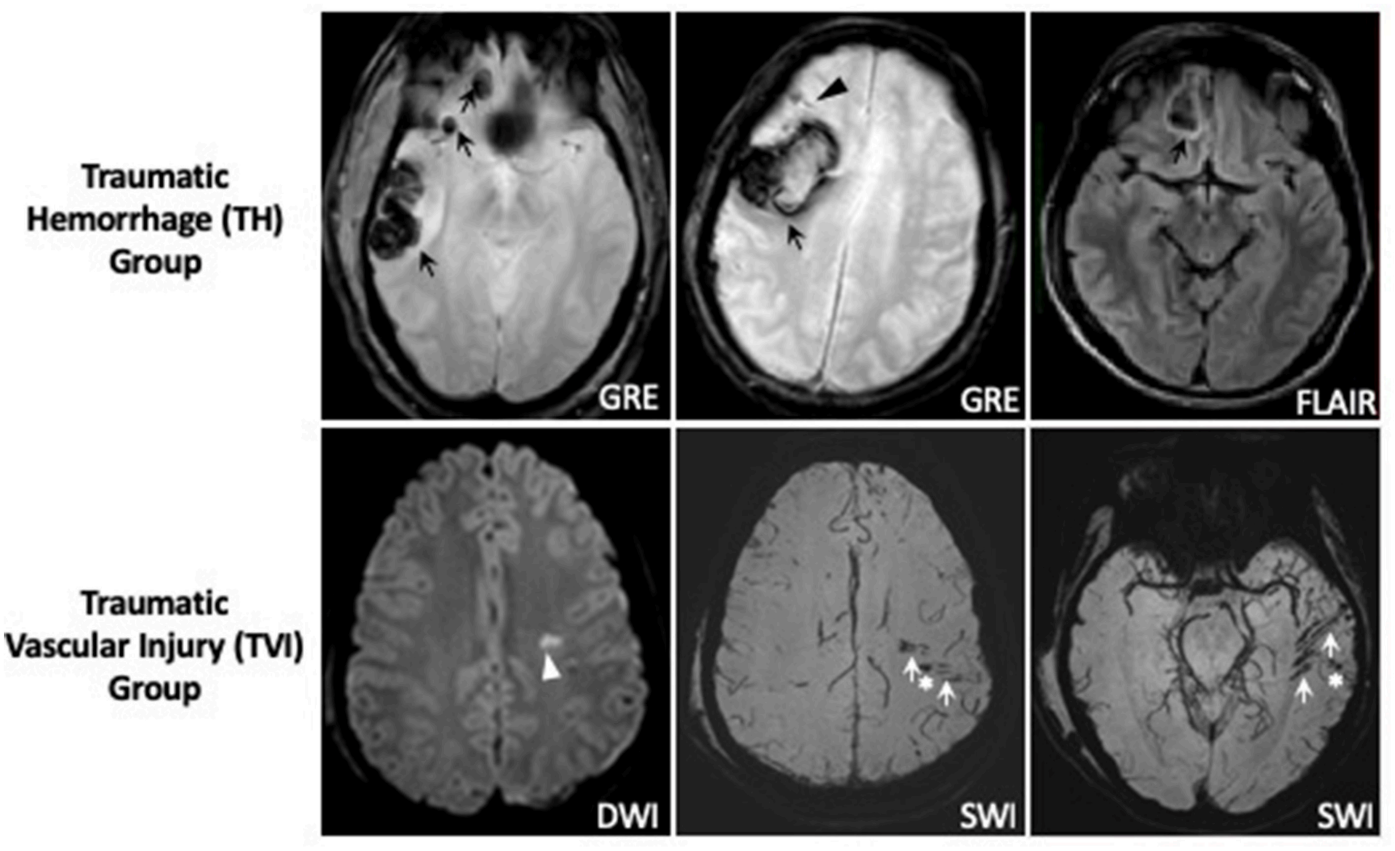
Representative MRI images of traumatic hemorrhage and traumatic vascular injury groups. Subjects classified in the TVI group had evidence of large (>1 cm) intraparenchymal (black arrows), subarachnoid (black arrowhead), subdural, or epidural hemorrhages on MRI sequences, including GRE and FLAIR sequences as shown. Subjects were classified in the TVI group if their MRI demonstrated one or more hyperintense lesions on DWI (white arrowhead) and/or presence of hypointense branching or linear structures on SWI (white arrows) or microhemorrhages <1 cm in size (white star*) in the absence of >1 cm intraparenchymal, subarachnoid, subdural, or epidural hematomas.

A total of 76 patients with TBI who underwent MRI scanning within 48 h of injury were included in this analysis (Figure 1). Most patients had mild injuries, as defined by a GCS score of 13–15 at hospital admission (Table 1). All subjects had head CTs at the time of their injuries; 23 patients had abnormal head CTs with findings of acute TBI injuries (subdural, subarachnoid, epidural, or intraparenchymal hemorrhages) during their initial evaluation. Subjects were classified into groups based on their predominant MRI findings (Figure 2). Thirty TBI patients did not have findings of TBI-associated injury on MRI (MRI-negative group). A second group, defined as the Traumatic Hemorrhage (TH) group (*n* = 26) had classic MRI findings of TBI, including IPH, EDH, SAH, and SDH. A third group (*n* = 20) did not have IPH, SDH, or SAH detected on MRI but did have MRI imaging abnormalities including (1) presence of hypointense, branching or linear structures on susceptibility weighted imaging, (2) presence of microhemorrhages (<1 cm) on GRE or SWI imaging, or (3) presence of one or more hyperintensities on diffusion weighted imaging [Traumatic Vascular Injury (TVI) group; Figure 1]. These three, MRI-defined groups were used for the subsequent analyses.

**Table 1:** Demographics of study participants.

	**MRI-negative (*n* = 30)**	**Traumatic hemorrhage (*n* = 26)**	**Traumatic vascular injury (*n* = 20)**	**ANOVA/Kruskal-Wallis**
**DEMOGRAPHICS**				
Age, years; median (IQR)	36 (27–57)	57 (45–72)	35 (28–55)	***p*** **=** **0.007**
% Male	57%	73%	90%	*p* = 0.17
Race: White	20 (67%)	21 (81%)	13 (65%)	*p* = 0.49
**INJURY CHARACTERISTICS**				
**Admission GCS**				*p* = 0.06
13–15	28 (93%)	21 (81%)	19 (95%)	
9–12	0 (0%)	1 (4%)	0 (0%)	
≤ 8	1 (3%)	4 (15%)	1 (5%)	
Unavailable	1 (3%)	–	–	
+ Loss of consciousness (%)	21 (70%)	14 (54%)	16 (80%)	*p* = 0.76
+Head CT on admission	0 (0%)	19 (73%)	3 (15%)	***P*** **<** **0.0001**
**CLINICAL OUTCOMES**				
GOS-E median (IQR)	*n* = 30 (100%) 7 (6–8)	*n* = 26 (100%) 7 (5-8)	*n* = 16 (80%) 6 (4.5–7.5)	***p*** **=** **0.02**
% good recovery (GOS-E 7-8)	83%	58%	50%	***p*** **=** **0.04**

*Bold indicates statistical significance of P < 0.05*.

The three groups of patients were similar in terms of demographic and injury/clinical characteristics (Table 1). TBI subjects that had traumatic hemorrhages (TH) were slightly older than MRI-negative or TVI subjects. The groups had similar clinical outcomes as determined by the GOS-E (Table 1).

### vWF, a Biomarker of Vascular Injury, Is Altered in Individuals With TBI-Related Hemorrhage or Vascular Injury

**Figure 3:**
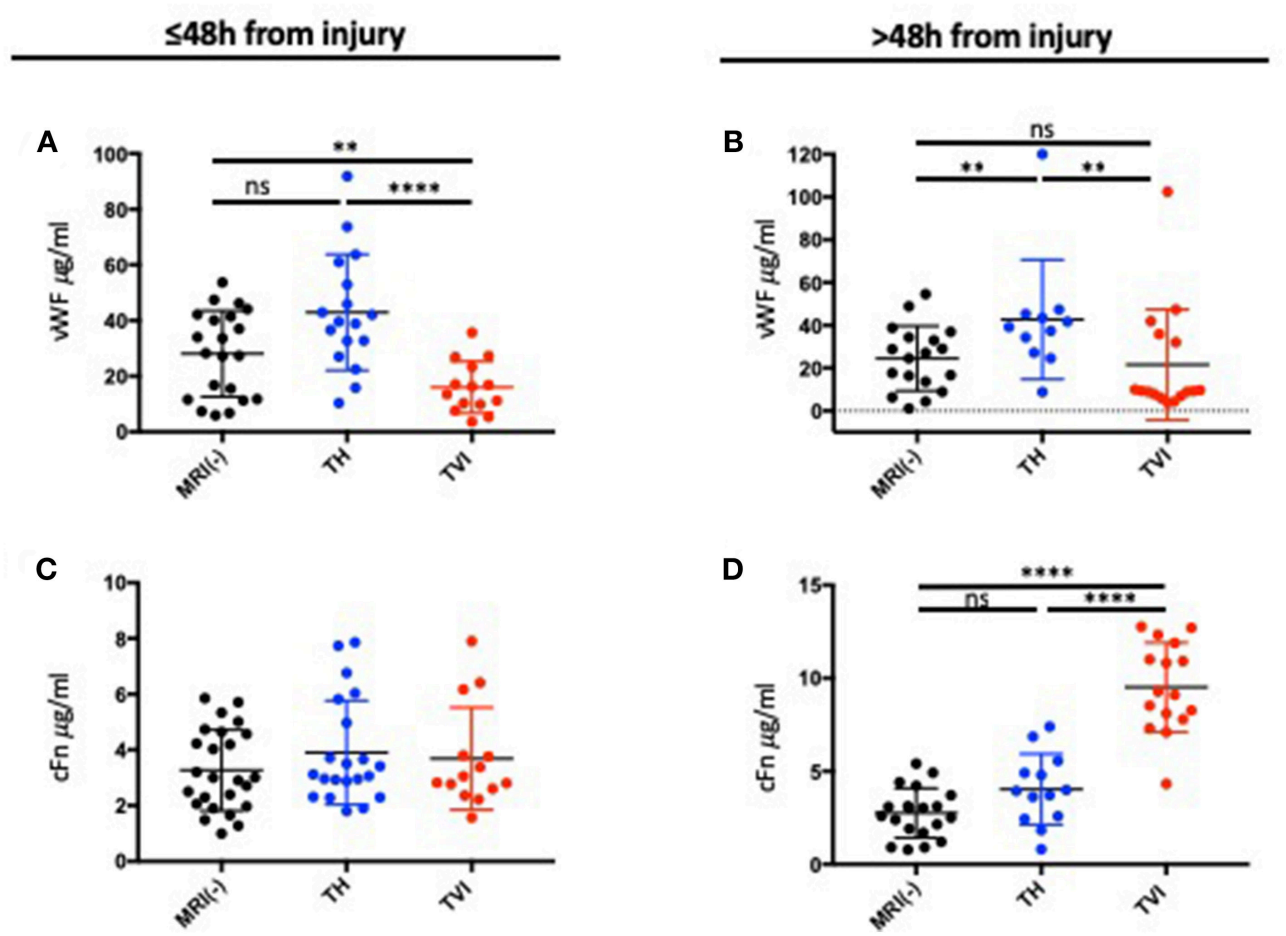
Plasma expression of vWF and cFN following TBI. **(A)** Plasma vWF measured ≤48 h after injury and **(B)** >48 h after injury. Plasma vWF was highest in the TH group and was decreased in the TVI group compared to the MRI-negative group at both time points. **(C)** Plasma cFn measured ≤48 h after injury and **(D)** >48 h after injury. There were no differences between the 3 MRI groups at the early time point. cFN was significantly increased in the TVI group >48 h after injury. Mann-Whitney tests were used to compare means between groups. ****p ≤ 0.00001; **p ≤ 0.05; ^ns^*p* ≥ 0.05 (non-significant).

We measured plasma biomarkers at ≤48 h from injury and at >48 h after injury. Less than 48 h after injury, vWF was increased in the TH group compared to the MRI-negative group (Figure 3A). vWF was lowest in the TVI group. These differences persisted in those patients in whom vWF could be measured >48 h from injury (Figure 3B).

### cFN Is Altered in Individuals With TBI-Related Vascular Injury, but Not Acute Traumatic Hemorrhage

c-Fn did not vary between groups <48 h from injury (Figure 3C), but was significantly elevated in the TVI group when measured **>**48 h after injury (Figure 3D) and distinguished this group from the MRI-negative and TH groups. cFN was not altered in TH subjects compared to MRI-negative subjects.

### pNF-H Is Elevated in Subjects With Abnormal MRI Scans After TBI

**Figure 4:**
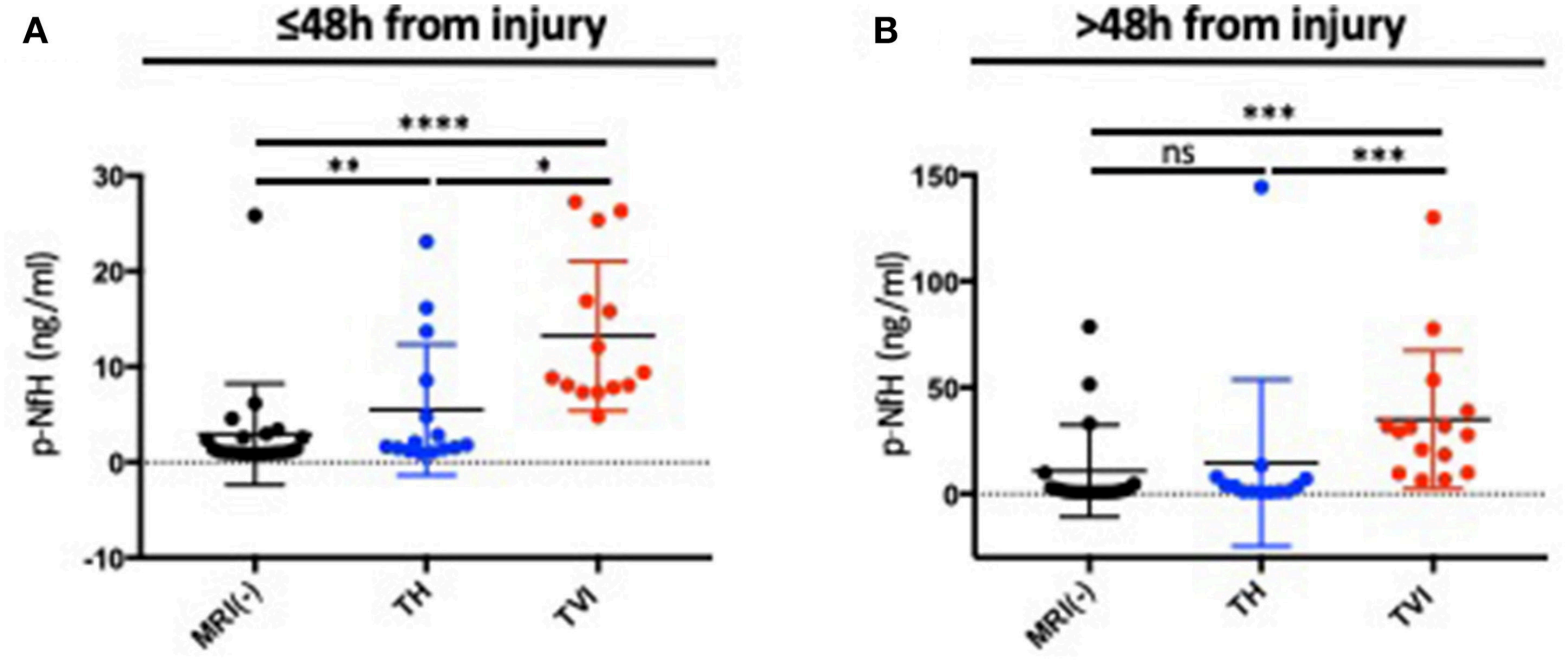
pNF-H expression distinguishes subjects with TBI-related MRI abnormalities. Plasma pNF-H was measured **(A)** ≤48 h after injury and **(B)** >48 h days after injury in the MRI(-), TH, and TVI groups. Both the TH and TVI groups had significantly higher levels of plasma pNF-H ≤48 h from injury than the MRI-negative group. The TVI group had higher levels of pNF-H at both time points. Mann-Whitney tests were used to compare means between groups. ****p ≤ 0.00001; ^ns^*p* ≥ 0.05; *p < 0.05; ** p< 0.01; *** p< 0.001.

In the acute period, pNF-H was increased in the both the TH and TVI group as compared to the MRI-negative group (Figure 4A). This effect persisted when measured **>**48 h after injury in the TVI group, though there was no longer a statistically significant difference between the MRI-negative and TH groups (Figure 4B).

### vWF and cFN Expression Does Not Correlate With pNF-H Expression Following TBI

To determine if biomarkers of vascular injury (vWF and cFN) correlate with pNF-H, Spearman correlations were performed. vWF and cFn were moderately correlated in the first 48 h after injury (*r* = 0.387, *p* = 0.005), but not when measured >48 h after injury (*r* = 0.138, *p* = 0.323). The marker of axonal injury, pNF-H was not correlated with the vascular biomarker vWF in the first 48 h after injury (*r* = −0.107, *p* = 0.454), or at the later time point (*r* = −0.023, *p* = 0.872). Similarly, p-NfH did not correlate with levels of cFn <48 h (*r* = −0.012, *p* = 0.939) or >48 h (*r* = 0.013, *p* = 0.930) after injury.

### Biomarker Analysis Improves Prediction of Clinical Recovery

We wanted to determine if vWF, cFN, and pNF-H might help with prediction of clinical outcomes when combined with demographic and injury information (Table 2). The outcomes were dichotomized as moderate to severe disability (GOSE 1-6) or good recovery (GOSE 7-8) and logistical regression analysis performed. Using multivariable logistic regression analysis, we found that standard clinical variables (Age, sex, and GCS) were able to predict a good recovery slightly better than chance (57–60%). Adding in the MRI data increased the ability to accurately predict a good outcome (MRI+ vs. MRI–: 70% accurate prediction; TVI+ vs. TVI–: 73% prediction of good outcome), When levels of vWF, cFn, and pNF-H measured at <48 h from injury were added to the model, the ability to predict the likelihood of good recovery was increased (MRI+ vs. MRI–: 77%; TVI+ vs. TVI–: 81% accurate prediction). The same protein biomarkers measured >48 h after injury were slightly less informative (MRI+ vs. MRI–: 76%; TVI+ vs. TVI–: 74% accurate prediction), but still added value over clinical variables alone.

**Table 2:** Prediction of 30–90 day GOSE using clinical and imaging/molecular biomarkers.

**Prediction of 3Prediction of 30–90 day GOSE**	***R*^**2**^ (Nagelkerke)**	**Significance (Hosmer-Lemeshow)**	**Correctly classified good outcome (GOSE 7-8) (%)**
Age group	0.05	0.16	57
Age group, sex	0.06	0.65	59
Age group, *sex*, GCS	0.15	0.69	62
Age group, *sex, GCS*, MRI+/-	0.25	0.74	70
Age group, *sex, GCS*, MRI*+/-*, acute biomarkers	0.38	0.75	77
Age group, *sex, GCS*, MRI*+/-*, subacute biomarkers	0.25	0.52	76
Age group, *sex*, GCS, group, TVI+/-	0.23	0.93	73
Age group, *sex*, GCS, group, TVI*+/-*, acute biomarkers	0.44	0.38	81
Age group, *sex*, GCS, group, TVI*+/-*, subacute biomarkers	0.31	0.38	74

*Age groups: < 40, 40.60, and 60 years. MRI+/-:Negative group includes all subjects that did not have any evidence of trauma-related injury on MRI (30 patients). The MRI+ group includes all subject who had evidence of TBI-related abnormalities on MRI (both TH and lVIgroups). lVI+/-:lVI+ groups includes all subjects with isolated evidence of SWI vascular abnormalities without large intracranial hemorrhages (20 subjects). The lVI- group includes the MRI(-) (30 subjects) and TH groups (26 subjects)*.

## Discussion

In this study, we show that traumatic vascular injury, as defined using standard MRI techniques, can be distinguished from other MRI patterns of injury using plasma-based measurements of vWF, cFn, and pNF-H. We show that the addition of these biomarkers can improve the ability to predict favorable patient outcomes over traditionally used demographic and clinical variables. These data suggest that there is a distinct, vascular-predominant endophenotype in a subset of patients who sustain a TBI that is characterized by a distinct MRI and biomarker profile.

Though MRI is not routinely done in the acute setting after mild TBI, prior work has demonstrated that MRI is more sensitive to predicting outcome at 3 months (31, 32). Our MRI findings, done in the acute setting <48 h from injury, indicate that there are a subset of patients who have a predominantly microvascular pattern of injury seen on SWI or DWI imaging without associated contusion or intracranial hemorrhage. In more severely injured patients, the SWI/DWI imaging patterns we observed would generally be classified as diffuse axonal injury. It is more precise to refer to these microhemorrhages as TVI, since it remains to be shown that these lesions correlate with direct measures of axonal injury using techniques such as diffusion tensor imaging (DTI) or physiological measures of axonal function. The significance of these MRI findings in mildly injured patients, like the majority of subjects in this study, is unclear, but hemorrhagic lesions identified by MRI are associated with poorer outcome even in mild injuries (31). Pathological studies would suggest that both axonal and vascular injury result from diffuse shearing forces (14). Recently, we have shown that disruptions in cerebral vascular reactivity persist in chronic TBI (33). Identifying these deficits in the acute period may be particularly helpful to select those patients who may most likely to benefit from vascular-directed therapies.

Ultimately, a blood-based biomarker that correlates with pathophysiological mechanisms would be helpful to identify specific TBI endophenotypes and to predict and track recovery after injury. We examined biomarkers of vascular injury (vWF and cFN) and axonal injury (pNF-H) to see if expression levels of these proteins could distinguish these groups. We hypothesized that these vascular injury biomarkers would follow a distinct expression pattern in the TVI group. vWF factor is a blood glycoprotein required for normal homeostasis that is released by endothelial cells, with a smaller contribution from platelets (34), and is a marker of endothelial injury (35). vWF expression was highest in the TH group in both the acute and subacute time periods. This is perhaps not surprising given that there is obvious blood-brain barrier and endothelial disruption leading to radiologically evident hemorrhages in these cases. De Oliveira et al. (26) examined vWF levels in individuals with severe TBI and found that an increased level of vWF was associated with poorer clinical recovery. Unexpectedly, we found that vWF is decreased in the acute period after injury in individuals with TVI. These data are consistent with a prior report which found higher levels of plasma vWF in subjects with focal TBI, while lower levels were indicative of a more diffuse injury pattern (25). Our subjects with TVI do seem to have more diffuse injuries than those with focal lesions, but how this accounts for the differences in circulating vWF is not known. Variable levels of vWF expression have been reported in other vascular insults, like myocardial infarction, cardiac arrest, and stroke (35–37). vWF remained elevated in the TH group which is consistent with prior studies that reported a lasting derangement in vWF expression (25). After the first 48 h following injury, we did not find a significant difference in vWF expression in the TVI group compared to MRI(–) individuals.

We also looked at cFn, a multimeric glycoprotein found in the extracellular matrix that participates in vascular morphogenesis, angiogenesis, and wound healing (28). cFn is expressed between endothelial cells and pericytes to promote blood brain barrier integrity, and thus is thought to serve as a biomarker of microvascular basal lamina injury (38). It has been used as a marker hemorrhagic transformation following thrombolysis in acute ischemic stroke (39) and correlates with injury severity after TBI (27). cFN remained low in the first 48 h but was elevated in the TVI group when measured 48 h to 7 days after injury. The TH and MRI-negative groups, by contrast, exhibited similar expression of cFN at both time points. Copin et al. similarly observed increases in cFN in 24–48 h following TBI (27). This group also found that higher levels of cFN in their cohort of severely injured patients were associated with increased mortality, longer ICU stay, and decreased return to consciousness, but not wourse GOS-E outcomes at 3 months. Our TVI group, in which cFN levels were highest at >48 h after injury, had worse outcomes, as determined using the GOS-E, at 30–90 days. These data suggest that higher plasma levels of cFN indicate injuries associated with poorer outcomes.

We compared these vascular injury biomarkers with pNF-H. Neurofilaments are among the most abundant protein components of neurons (28). As these proteins are only expressed by neurons, their presence in the blood is indicative of neuronal damage and/or death following brain injury (29). pNF-H is relatively protease resistant, allowing it to be detected in clinical sample (40) and its detection in the blood is not related to blood-brain barrier dysfunction (41). Prior studies have shown that expression of pNF-H correlated with imaging findings of axonal injury and clinical recovery in children who had sustained a TBI (40, 42). Surprisingly, we found that plasma pNF-H levels were higher in the TVI group as compared to the TH and MRI(–) groups, both in the acute and subacute time periods. While the extent of intracranial hemorrhage seen on CT is frequently used as a surrogate of injury severity, these data suggest that degree of hemorrhage may not adequately reflect the extent of neuronal or vascular injury, a finding that is consistent with the poor correlation between neuroimaging findings and clinical symptoms reported by TBI patients (43–45). Taken together, these data suggest a model wherein TH and TVI are distinct injury patterns, and not simply a continuum of bleeding diathesis, with TVI representing a more “mild” bleeding injury than TH. This is consistent with data from our group showing that, in the chronic state, there can be cerebral vascular dysfunction even in the absence of frank injury from a TBI-related hemorrhage (33, 46).

Knowing that clinical and imaging characteristics are not particularly helpful in prognosticating overall recovery after TBI, particularly mild TBI, we sought to determine if inclusion of these biomarkers would be helpful to predict long-term recovery. We found that the inclusion of these biomarkers improved our ability to predict clinical recoveries, using the GOS-E as the outcome metric, over clinical data only.

This study has several limitations. It is based on a small sample size and individual groups (MRI-negative, TH, and TVI) are small. We did not have samples of age matched healthy volunteers. The study was designed to examine MRI markers of injury, and thus not all subjects had biomarker testing at both time points, the timing of biomarker testing was variable, and outcome surveys were not available on all subjects. Our TVI group was the smallest and a number of those subjects had missing outcome data which may skew these results. There is no consensus in the field regarding which biomarkers are best to measure TBI-related injury or at which timepoint they should be measured (47, 48). There are other vascular and axonal biomarkers which we could have examined, but sample volumes were limited. These findings will need to be replicated in a larger sample set, which we are currently collecting.

Overall, our data suggest that there is a distinct, vascular-predominant endophenotype in a subset of patients who sustain TBI and that these injuries are characterized by a distinct vWF, cFN, and pNF-H profile. Further work is needed to better characterize the exact timeline of these expression changes and how they correlate with other imaging metrics of vascular and axonal injury, such as cerebrovascular reactivity measurements and diffusion tensor imaging. These data provide important first insights into how we might be able to classify TBI disease subtypes to better select patients for targeted therapeutics.

## Data Availability

The datasets generated for this study are available on request to the corresponding author.

## Author Contributions

LL, RD-A, and TB contributed to the conception and design of the study. MC, CD, and JB assisted with study enrollment, imaging acquisition, and data analysis. B-XQ and TB performed biosample analysis. DS, MH, and TB performed statistical analyses. DS and TB wrote the manuscript. All authors contributed to the manuscript review and revision and approved the submitted version.

### Conflict of Interest Statement

TB is currently employed by Elsai, Inc. The work contained in this manuscript was prior to this affiliation. The remaining authors declare that the research was conducted in the absence of any commercial or financial relationships that could be construed as a potential conflict of interest.
